# Neurodevelopmental and environmental hypotheses of negative symptoms of schizophrenia

**DOI:** 10.1186/1471-244X-14-88

**Published:** 2014-03-26

**Authors:** Frédéric Limosin

**Affiliations:** 1 Department of Adult and Geriatric Psychiatry, Hôpitaux Universitaires Paris Ouest (AP-HP), Hôpital Corentin-Celton, 4, parvis Corentin-Celton, 92133 Issy-les-Moulineaux, France; 2 Faculty of Medicine, Université Paris Descartes, Sorbonne Paris Cité, Paris, France; 3 Psychiatry and Neurosciences Center, French National Institute of Health and Medical Research (Inserm) U894, 75014 Paris, France

**Keywords:** Schizophrenia, Negative symptoms, Environment, Neurodevelopmental hypothesis

## Abstract

The negative symptoms of schizophrenia, avolition, alogia, apathy and impaired or nonexistent social functioning, are strongly correlated with the progressive course and long-term prognosis of the disease, undermining the patient’s ability to integrate socially, interpersonal skills and quality of life. At a time when new drug strategies are being developed, a better understanding of the etiology and pathogenesis underpinning the occurrence of negative symptoms constitutes an essential prerequisite for real therapeutic advances. Approaching this vulnerability from the neurodevelopmental perspective is especially pertinent with regard to the experimental studies conducted in animals. Several models have been put forward, involving a variety of topics such as the deleterious impact of a prenatal infection or of early maternal deprivation on brain development, or else the consequences of trauma and abuse suffered during childhood. These various models are based on biological abnormalities that could guide the identification of new therapeutic targets. They notably include the hyperreactivity of the hypothalamic-pituitary-adrenal axis and dysfunction of corticostriatal glutamatergic transmission. As such, in the traumagenic model, which associates neurodevelopmental and neurodegenerative processes, the dysfunction of corticostriatal glutamatergic transmission, by reducing the tonic dopamine release, could be the cause of an increase in the phasic dopamine release linked to stress. This excessive phasic response to stress may induce cerebral damage by increasing excitotoxicity and oxidative stress.

## Introduction

The complexity and clinical heterogeneity of schizophrenia raise questions about the respective roles of the various vulnerability factors involved. Among these factors, those related to the environment, whether of a psychosocial or biological nature, whether occurring early or later, remain non-specific factors that may play a role in the etiology and course of the disorder.

The neurodevelopmental hypothesis is founded on evidence from clinical, experimental and brain imaging findings. This model is based on the occurrence of early abnormalities in neurodevelopmental processes, well in advance of the onset of the disorder. The main neurodevelopmental hypotheses implicate structural abnormalities involving the migration of neurons and laminar differentiation of the cortex, occurring during the second trimester of pregnancy. However, all stages of normal brain development may be involved, from the proliferation of stem cells to the differentiation and migration processes, and then from dendritic and axonal growth to the myelination process, a process that continues at least until early adulthood, notably in the prefrontal cortex.

## Review

Epidemiological studies of separation/adoption, of half-siblings and twins show that the familial clustering of cases of schizophrenia is not solely the result of genetic factors, but also of environmental factors. Twin studies estimate heritability at approximately 80% [1], while Lichtenstein et al., in a register-based study of Swedish families, determined that the genetic factors accounted for 64% of the vulnerability to develop schizophrenia, compared to 36% for environmental factors [2]. Even if the respective influence of these two types of vulnerability factors varies according to the disease subtype (hence the importance of distinguishing disease phenotypes), their interaction appears to be fundamental to the etiology and course of the disorder. In addition, the notion of vulnerability and the role of environmental factors are complex notions in more than one respect:

1. Firstly, the influence of environmental factors can be exerted on the occurrence of the disorder, by contributing to the underlying etiology, as well as on its progressive course, whether this be in terms of clinical characteristics (severity, age of onset, comorbidities, etc.), or in terms of the response to drug treatments.

2. Secondly, the notion of environment encompasses highly varied factors, both by their nature (demographic, climate, dietary, psychological, etc.) and by the stage of development at which they intervene. As such, ‘early’ environmental factors, whose influence is exerted well in advance of the onset of the disease, such as pre-and peri-natal factors, are distinguished from ‘late’ factors, such as, for example, the role of stressful life events in the progressive course of the disorder. Ultimately, the environment is understood in a very broad, and thus heterogeneous sense, ranging from the internal biological environment to the external context.

3. Lastly, we can no longer consider environmental factors in isolation but rather must consider them within integrated vulnerability models. In addition, more so than causal risk factors, vulnerability factors are considered to be predisposing disease factors or intermediate factors whose influence alone would be insufficient to trigger the onset of the disorder. In these models, the interactions between environmental factors and genetic factors appear to be especially relevant.

## Environmental factors most often implicated

### Psychosocial factors

• The consequences of *immigration* have given rise to several epidemiological studies, notably in England and the Netherlands, which found an increased risk of developing schizophrenia in immigrants, whether of the first or second generation [3]. This risk could be partly due to conditions of greater social adversity, but other hypotheses have been put forward, such as the mother’s diminished immunity to the viruses of the host country.

• *The psychological effect of an urban environment* may also interact with levels of reality distortion and depressive symptoms in people experiencing first-episode psychosis and may affect the syndromal presentation of psychotic disorders [4]. For individuals suffering from psychosis, spending time in an urban environment makes them think more negatively about other people and increases their paranoia [5].

• The role of *disturbances in early parent/child relationships*, whether it be early separation, a lack of emotional support and physical contact, or abuse, has also been invoked in the later development of the disorder. Zugno et al. showed that rats subjected to maternal deprivation had an increased risk for schizophrenia-like behavior and cholinergic alteration [6].

### Biological factors

• *Toxicity factors*, foremost among them, psychoactive substances like cannabis, amphetamines or cocaine, can cause acute episodes punctuating the course of the disorder. In most cases, the toxic agents involved determine to a lesser extent the onset of a chronic disease than they precipitate its onset or interfere with its progressive course, making it worse with more complications and sometimes poorer treatment response [7]. Nonetheless several prospective cohort studies on the general population have shown that subjects who use cannabis had a higher risk of developing a psychotic disorder [8].

• *Role of certain infectious agents:* It was during the nineteenth century that the hypothesis of the role of infectious agents in the occurrence of schizophrenia, as well as in bipolar disorder, was first formulated, notably by Jean Esquirol in 1845, followed by Eugen Bleuler in 1911 and Emil Kraepelin in 1919 [9]. This hypothesis was in line with the discovery of the infectious nature of the psychiatric manifestations of syphilis which occurred during the eighteenth century. Among the different infectious agents, the influenza virus has been implicated most often. Its pathogenic role was suggested for the first time during the 1918 pandemic: many individuals who contracted the "Spanish" flu developed psychiatric disorders, especially with clinical presentations that resembled schizophrenia, as described at the time by Menninger [10].

However, the epidemiological starting point for the hypotheses formulated on the role of early environmental vulnerability factors was the demonstration of a seasonal imbalance in the distribution of births of future patients, which is evident in schizophrenia. Many studies have identified, in the Northern Hemisphere, an excess of births of subjects with schizophrenia during the winter and early spring, especially between December and April [11]. On average, it is estimated that in European and North American countries, the proportion of subjects with schizophrenia born in winter or at the start of spring exceeds the figures expected in the general population by about 5 to 15%. The "age-incidence" effect, which concerns all diseases where the incidence increases with the age of subjects, seems too small to account for the observed seasonal peak of births. In addition, the existence of this peak during the austral winter in the Southern Hemisphere, even though it is less evident, runs counter to such a bias [12]. This phenomenon of seasonal imbalance is not specific to schizophrenia, since it is found in other diseases like bipolar disorder [13] or substance or alcohol abuse [14].

In order to account for the observed seasonal distribution of births, various hypotheses have been put forward, some of which are more satisfactory than others: climate, dietary, obstetrical, chronobiological or infectious. Among these alternatives, two quickly gained traction: the role of obstetrical complications and the infectious hypothesis, falling in line with a neurodevelopmental model of schizophrenia.

• *Role of obstetrical complications:* obstetrical complications are associated with an increased risk of developing schizophrenia. It is a well-documented yet low risk with an odds ratios adjusted for the effect of exposure being approximately 2. The complications most often involved are bleeding during pregnancy, pre-eclampsia and gestational diabetes, as well as certain delivery complications, such as uterine atony, neonatal asphyxia and hemorrhage [15]. Once again, this association is not specific to schizophrenia, as a higher frequency of obstetrical complications has been found in other psychiatric disorders, such as mood disorders, anorexia nervosa or autism [16,17].

• *Role of the influenza virus:* influenza infection during the gestation period is associated with an increased risk for the child of later developing schizophrenia [18]. Once again, this association is not specific to this diagnosis, as it is found, for example, in the predisposition to unipolar mood disorder [19]. The mechanism of action of the influenza virus has not been elucidated, but the leading hypothesis suggests that in certain genetically predisposed mothers, antiviral maternal antibodies could cause an autoimmune reaction which is harmful to the fetal brain [20].

## The neurodevelopmental hypothesis applied to negative symptoms

The negative symptoms of schizophrenia include avolition, alogia, apathy and impaired or nonexistent social functioning. The negative symptoms are so called because they are an absence as much as a presence: inexpressive faces, monotone and monosyllabic speech, few gestures, seeming lack of interest in the world and other people, inability to feel pleasure or act spontaneously. Origin of the definition of the negative symptoms can be found in the work of John Hughlings Jackson [21]. From 1874 to 1876, Hughlings Jackson adapted Herbert Spencer's evolutionary theory to neurological disease and addressed the relationship between the centres in his hierarchy. Following Spencer, Hughlings Jackson concluded that the highest nervous centres evolved out of the lower. Patients with diseases of the highest centres develop two types of symptoms, negative symptoms due to the loss of higher centres and positive symptoms due to the emergence of lower centres. Positive symptoms are simpler and less differentiated than the negative symptoms which they replace [21]. Furthermore, recent data suggest that negative symptoms should not be considered only as a unitary construct, and that measurement of these symptoms should distinctly assess motivation, pleasure, and emotion expression factors [22]. Moreover, particular attention should be paid to the distinction between primary versus secondary negative symptoms and between prominent and predominant negative symptom profiles [23].

The frequency of the deficit syndrome varies depending on the progressive stage of the disease, from about 15% for the first episodes up to 25-30% for the later phases [24]. The wider use of second-generation antipsychotics may have permitted an appreciable reduction in the prevalence of negative symptoms; at least, this is what the recent study by Shivashankar et al. [25] suggests, finding such a reduction over a period of 25 years (between 1981 and 2006). The fact nevertheless remains that negative symptoms comprise a major factor in interpersonal and social isolation and impairment of quality of life. Yet, even if negative symptoms are associated with greater genetic determinism than positive symptoms [26-29], the analysis of the neurodevelopmental factors likely to be involved in their occurrence appears to be relevant in more than one respect, especially from the perspective of identifying new therapeutic targets.

### Data from epidemiological studies

Several studies have demonstrated an association between prenatal infection and certain functions and morphological brain abnormalities in patients with schizophrenia, whether they be in executive function deficits or an enlarged cavum septum pellucidum, suggestive of dysgenesis [30,31].

Some studies have found an association between the peak of birth seasonality and certain clinical forms, mainly disorganized subtype [32], paranoid subtype [33] or with pronounced negative symptomatology [34]. In addition, patients with schizophrenia who were exposed to an elevated risk of influenza infection during pregnancy are more likely to be single, which could indicate poorer social adjustment [35], and with the disorganized clinical subtype [18].

### Experimental studies conducted in animals

• Prenatal infection model

  One of the experimental models used to analyze the impact of a prenatal infection consists of injecting the pregnant female rodent with polyriboinosinic-polyribocytidylic acid (Poly I:C), a synthetic analog of double-stranded RNA that stimulates an immune response with cytokine secretion. It has been shown that this exposure is the cause of cognitive, behavioral and pharmacological dysfunction in the neonate as an adult, and that these abnormalities result in disorders found in schizophrenia, such as disorders of sensorimotor gating, selective attention, working memory or hypersensitivity to psychostimulants.

  In addition, the timing of this exposure appears to be paramount, since the abnormalities induced will vary, depending on the stage of pregnancy. Thus, if exposure is late, at the end of gestation, one observes as an adult rodent persistent behavioral abnormalities with learning disabilities [36], disturbance of visual-spatial working memory [37] and hypersensitivity to the glutamatergic NMDA (N-methyl-D-aspartate) receptor blockade during treatment with the antagonist dizocilpine (MK-801) [37]. They thus involve precisely the dysfunctions that may underpin the negative symptoms found in schizophrenia.

  Bitanihirwe et al. [38] injected mice with Poly I:C on gestation day 17, hence during the late gestational phase. In those neonates grown as adults, deficits in social interaction, anhedonic behavior, and alterations in locomotor behavioral response to acute administration of apomorphine were demonstrated. In addition, male subjects were found to lack cognitive and behavioral flexibility. Lastly, the authors found reduced levels of dopamine and glutamate in the prefrontal cortex (PFC) and hippocampus, as well as decreased levels of γ-aminobutyric acid (GABA) in the hippocampus and of glycine in the PFC. These abnormalities are in favor of a neurodevelopmental model that could account for the occurrence of negative symptoms, with, in addition, an effect correlated with gender.

• Experience of maternal deprivation

  In the neonate rat, long periods of maternal deprivation (from 3 to 24 hours versus 3 to 15 minutes) are associated with hyperactivity of the HPA (hypothalamic-pituitary-adrenal) axis [39,40], and with a prepulse inhibition (PPI) deficit or lack of reduction in the intensity of startle [41]. The term PPI describes the inhibition of the startle reflex triggered by a violent and unexpected exteroceptive stimulus (pulse) when it is preceded in the short term by a weaker prestimulus (prepulse), too weak to itself induce the reflex. PPI is thus an operational measure of sensorimotor gating, reflecting the capacities of the individual to regulate, to "buffer" the input of different levels of significance and variable chaotic flow of sensory information. Maternal deprivation is also accompanied by cognitive disorders with impaired learning abilities [42,43].

  Moreover, there is a period of brain development particularly sensitive to the deleterious effects of maternal deprivation: the stress hyporesponsive period, which falls, for the rat, between the 4th and 14th postnatal day. Takase et al. [44] thus showed that among rats subjected to maternal deprivation during this critical period, only the postpubertal males had abnormal hippocampal calcineurin expression associated with a deficit in social interactions and objective memory. Once again, this is an animal model linking negative symptoms and neurodevelopmental impairment.

### The traumagenic neurodevelopmental model

This model was proposed in light of two observations: that of the high prevalence of childhood abuse in patients with schizophrenia [45], and that of the similarities between the effects on brain development of the trauma from abuse and certain biological abnormalities found in patients with schizophrenia, whether it be hyperreactivity of the HPA axis or morphological changes to the brain (cerebral atrophy, ventricular enlargement, reversed cerebral asymmetry, hippocampal lesions).

Ross et al. [46] showed that a history of abuse was negatively correlated with negative symptoms. However, there may be two distinct patterns of response to stress:

• Some subjects repeatedly confronted with abuse react through dissociative and positive symptoms: it then involves not so much a process of cerebral atrophy, as dysfunction mediated by dopamine, whence a better response to antidopaminergics [47].

• Other subjects react through hypervigilance (anxiety, hypersensitivity) that may accentuate the phenomenon known as "pruning", normally occurring at adolescence and corresponding to a decrease in the number of synapses (especially in the neuropil) and to neuronal loss. This excessive neuronal loss could be at the root of the progression toward negative symptoms in adulthood [48,49].

In this latter model, which associates neurodevelopmental and neurodegenerative processes, the glutamate hypothesis could also play a role. In fact, dysfunction of corticostriatal glutamatergic transmission, by reducing the tonic dopamine release, could be the cause of an increase in the phasic dopamine release linked to stress. This excessive phasic response to stress may induce cerebral damage by increasing excitotoxicity and oxidative stress.

Lastly, still in this mixed model associating a neurodevelopmental approach and a neurodegenerative process, some immune dysfunction could be involved, mainly implicating the role of pro-inflammatory cytokines [50].

## Conclusion

The negative symptoms of schizophrenia are a major factor in interpersonal and social isolation and the impairment of quality of life, and thus represent a major issue in the field of development of new therapeutic strategies, notably drug strategies. As such, a better understanding of the pathogenic mechanisms that underpin the occurrence of negative symptoms is an essential prerequisite for the identification of therapeutic targets. In this perspective, the neurodevelopmental approach appears particularly relevant, especially with regard to experimental animal studies, which have made it possible to demonstrate a number of biological abnormalities. These notably include hyperreactivity of the HPA axis and dysfunction of corticostriatal glutamatergic transmission. Additionally, given the modest impact of drug treatments for negative symptoms to date [51], psychological factors associated with the development of these symptoms are of considerable importance and may be viable treatment targets [52].

## Abbreviations

HPA: Hypothalamic-pituitary-adrenal; GABA: γ-aminobutyric acid; NMDA: N-methyl-D-aspartate; PFC: Prefrontal cortex; Poly I:C: Polyriboinosinic-polyribocytidylic acid; PPI: Prepulse inhibition; RNA: Ribonucleic acid.

## Competing interest

FL, in the last five years, received fees for advisory boards or symposia from AstraZeneca, Bristol-Myers Squibb, Eutherapie, Janssen, Lundbeck, Otsuka and Roche.

## Pre-publication history

The pre-publication history for this paper can be accessed here:

http://www.biomedcentral.com/1471-244X/14/88/prepub
